# Grape seed proanthocyanidin extract inhibits glutamate-induced cell death through inhibition of calcium signals and nitric oxide formation in cultured rat hippocampal neurons

**DOI:** 10.1186/1471-2202-12-78

**Published:** 2011-08-03

**Authors:** Seo-Hee Ahn, Hee Jung Kim, Imju Jeong, Yi Jae Hong, Myung-Jun Kim, Duck-Joo Rhie, Yang-Hyeok Jo, Sang June Hahn, Shin Hee Yoon

**Affiliations:** 1 Department of Physiology, College of Medicine, The Catholic University of Korea, 505 Banpo-dong, Seocho-gu, Seoul 137-701, Korea; 2 the Catholic Institute for Advanced Biomaterials, The Catholic University of Korea, 505 Banpo-dong, Seocho-gu, Seoul 137-701, Korea; 3 Department of Physiology, College of Medicine, Dankook University, San 29, Anseo-dong, Cheonan-si, Chungnam 330-714, Korea

## Abstract

**Background:**

Proanthocyanidin is a polyphenolic bioflavonoid with known antioxidant activity. Some flavonoids have a modulatory effect on [Ca^2+^]_i_. Although proanthocyanidin extract from blueberries reportedly affects Ca^2+ ^buffering capacity, there are no reports on the effects of proanthocyanidin on glutamate-induced [Ca^2+^]_i _or cell death. In the present study, the effects of grape seed proanthocyanidin extract (GSPE) on glutamate-induced excitotoxicity was investigated through calcium signals and nitric oxide (NO) in cultured rat hippocampal neurons.

**Results:**

Pretreatment with GSPE (0.3-10 μg/ml) for 5 min inhibited the [Ca^2+^]_i _increase normally induced by treatment with glutamate (100 μM) for 1 min, in a concentration-dependent manner. Pretreatment with GSPE (6 μg/ml) for 5 min significantly decreased the [Ca^2+^]_i _increase normally induced by two ionotropic glutamate receptor agonists, N-methyl-D-aspartate and alpha-amino-3-hydroxy-5-methyl-4-isoxazolepropionic acid (AMPA). GSPE further decreased AMPA-induced response in the presence of 1 μM nimodipine. However, GSPE did not affect the 50 mM K^+^-induced increase in [Ca^2+^]_i_. GSPE significantly decreased the metabotropic glutamate receptor agonist (*RS*)-3,5-Dihydroxyphenylglycine-induced increase in [Ca^2+^]_i_, but it did not affect caffeine-induced response. GSPE (0.3-6 μg/ml) significantly inhibited synaptically induced [Ca^2+^]_i _spikes by 0.1 mM [Mg^2+^]_o_. In addition, pretreatment with GSPE (6 μg/ml) for 5 min inhibited 0.1 mM [Mg^2+^]_o_- and glutamate-induced formation of NO. Treatment with GSPE (6 μg/ml) significantly inhibited 0.1 mM [Mg^2+^]_o_- and oxygen glucose deprivation-induced neuronal cell death.

**Conclusions:**

All these data suggest that GSPE inhibits 0.1 mM [Mg^2+^]_o_- and oxygen glucose deprivation-induced neurotoxicity through inhibition of calcium signals and NO formation in cultured rat hippocampal neurons.

## Background

Proanthocyanidins are polymers of flavonoid molecules that are widely available in fruits, vegetables, nuts, seeds, flowers, and bark, and especially in grape seeds [1]. These compounds possess a broad spectrum of antioxidative properties that provide potent protection against free radical-induced diseases, such as ischemia and reperfusion injury [2-4], aging [5], and carcinogenesis [6]. Proanthocyanidins are also known to possess antibacterial, antiviral, anti-inflammatory, anti-allergic, and vasodilator properties [1,7].

Glutamate is a major neurotransmitter in the central nervous system. Glutamate increases intracellular free Ca^2+ ^concentration ([Ca^2+^]_i_) in neurons by activating ionotropic and metabotropic glutamate receptors. In pathological conditions, including epilepsy and ischemia, a massive glutamate release leads to glutamate neurotoxicity [8,9]. The neurotoxicity is mainly due to N-methyl-D-aspartate (NMDA) receptors, which cause excessive elevation of intracellular Ca^2+ ^concentration ([Ca^2+^]_i_) and subsequent neuronal cell death [10]. Elevation of [Ca^2+^]_i _following NMDA receptor activation stimulates nitric oxide synthase (NOS), an enzyme that induces formation of nitric oxide (NO) in neurons [11]. NO reportedly also mediates glutamate neurotoxicity [12,13].

Some flavonoids have modulatory effects on [Ca^2+^]_i_. (-)-Epigallocatechine gallate (EGCG) increase [Ca^2+^]_i _in U87 cells [14] and inhibit glutamate-induced [Ca^2+^]_i _increase in PC12 cells [15] and cultured rat hippocampal neurons [16]. Quercetin has stimulatory effects on voltage-dependent L-type Ca^2+ ^channels in GH3 cells and inhibitory effects on L-type Ca^2+ ^channels in NG108-15 cells [17]. In addition, EGCG [15], apigenin [18], and wogonin [19] have a neuroprotective effect in glutamate neurotoxicity. Proanthocyanidin extract from blueberries has reportedly reversed dopamine, Aβ_42_, and lipopolysaccharide-induced dysregulation of Ca^2+ ^buffering capacity [20]. However, there are no reports on the effect of proanthocyanidin on glutamate-induced [Ca^2+^]_i _or cell death in cultured rat hippocampal neurons.

The present study determined whether grape seed proanthocyanidin extract (GSPE) affected glutamate-induced Ca^2+ ^signalling and NO formation in cultured rat hippocampal neurons. It further examined whether GSPE protects neurons against neurotoxicity induced by low extracellular Mg^2+ ^concentration ([Mg^2+^]_o_) and oxygen glucose deprivation.

## Results

### Effect of GSPE on glutamate-induced [Ca^2+^]_i _increase

**Figure 1:**
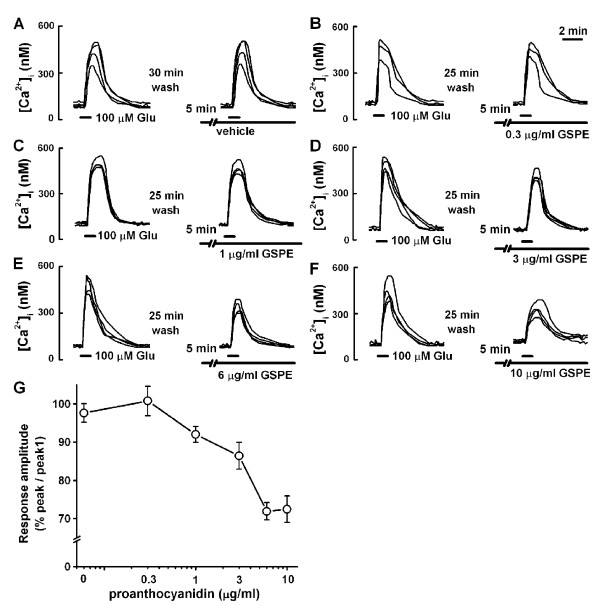
**GSPE inhibits glutamate-induced [Ca^2+^]_i _increase in cultured rat hippocampal neurons**>. A: Reproducible increase in glutamate-induced [Ca^2+^]_i _was induced by treatment with glutamate (100 μM) for 1 min at 30 min intervals. B-F: Pretreatment with GSPE for 5 min inhibited glutamate-induced response in a concentration-dependent manner. G: Plot summarizes the inhibition of GSPE on glutamate-induced [Ca^2+^]_i _increase (0.3 μg/ml, n = 15; 1 μg/ml, n = 17; 3 μg/ml, n = 16; 6 μg/ml, n = 21; 10 μg/ml, n = 16). Glutamate-induced response is presented as a percentage of initial glutamate-induced response (peak 2/peak 1) for vehicle and GSPE-pretreated cells. Data are expressed as the mean ± SEM.

Since elevation of [Ca^2+^]_i _is one of the major causes of glutamate excitotoxicity [10], the present study first examined the effect of GSPE on glutamate-induced [Ca^2+^]_i _increase in cultured rat hippocampal neurons. Treatment with glutamate (100 μM) for 1 min caused [Ca^2+^]_i _increase. Reproducible response could be elicited by applying glutamate (100 μM) for 1 min at 30-min intervals (peak 2/peak 1 = 97.6 ± 2.4%, n = 27) (Figure 1A). Pretreatment with GSPE (0.3 μg/ml) for 5 min did not affect the glutamate-induced [Ca^2+^]_i _response (peak 2/peak 1 = 100.8 ± 3.8%, n = 15) (Figure 1B). Pretreatment with higher concentrations of GSPE (1-6 μg/ml) inhibited the glutamate-induced response in a concentration-dependent manner (peak 2/peak 1 = 92.0 ± 2.1% at 1 μg/ml, n = 17; 86.5 ± 3.5% at 3 μg/ml, n = 16; 71.9 ± 2.3% at 6 μg/ml, n = 21). However, pretreatment with 10 μg/ml GSPE did not further inhibit the glutamate-induced response (peak 2/peak 1 = 72.4 ± 3.5% at 10 μg/ml, n = 16) (Figure 1C-G). Therefore, the present study used 6 μg/ml of GSPE to quantify the inhibition of agonist-induced [Ca^2+^]_i _increase. The 6 μg/ml concentration of IHEA GSPE used in the present study was less than or equal to the serum levels of polyphenols after intake of grape seed proanthocyanidin extract in humans [21].

### Effect of GSPE on ionotropic glutamate receptor agonist-induced [Ca^2+^]_i _increase

**Figure 2:**
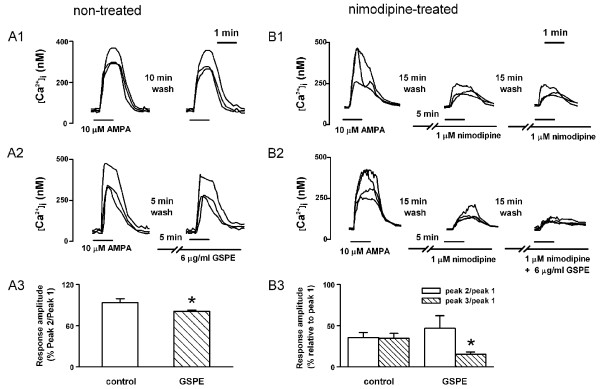
**GSPE inhibits AMPA-induced [Ca^2+^]_i _increase in the absence (A) or presence (B) of nimodipine**. A1: Reproducible AMPA-induced [Ca^2+^]_i _increase was induced by treatment with 10 μM (*S*)-AMPA for 1 min at 10 min intervals. A2: Pretreatment with GSPE (6 μg/ml) for 5 min decreased the AMPA-induced response. A3: The graph summarizes the effect of GSPE on AMPA-induced response (control, n = 20; GSPE, n = 23). B1: Pretreatment with 1 μM nimodipine for 5 min inhibited AMPA-induced [Ca^2+^]_i _response. B2: Pretreatment with GSPE (6 μg/ml) for 5 min further decreased AMPA-induced response in the presence of 1 μM nimodipine. B3: The graph summarizes the effect of GSPE on AMPA-induced response in the presence of nimodipine (control, n = 11; GSPE, n = 9). Data are expressed as the mean ± SEM. *P < 0.05 relative to the respective control (unpaired Student's *t*-test).

To determine how GSPE inhibits glutamate receptor-induced [Ca^2+^]_i _increase, the present study used two ionotropic glutamate receptor agonists, alpha-amino-3-hydroxy-5-methyl-4-isoxazolepropionic acid (AMPA) and NMDA. Application of AMPA increased [Ca^2+^]_i _by activating AMPA/kainate channels and then voltage-gated Ca^2+ ^channels in neurons. Reproducible increase in [Ca^2+^]_i _was induced by treatment with (*S*)-AMPA (10 μM) for 1 min at 10-min intervals (peak 2/peak 1 = 93.7 ± 5.4%, n = 20). Pretreatment with GSPE (6 μg/ml) for 5 min significantly inhibited the AMPA-induced [Ca^2+^]_i _response (peak 2/peak 1 = 80.8 ± 2.0%, n = 23, *P *< 0.05) (Figure 2A1-A3).

Ca^2+^-permeable AMPA receptors are expressed in hippocampal neurons early in development [22]. The present study tested whether proanthocyanin inhibits Ca^2+^-permeable AMPA-receptor-mediated Ca^2+ ^influx. Pretreatment with nimodipine (1 μM) for 5 min inhibited the AMPA-induced [Ca^2+^]_i _response (peak 2/peak 1 = 40.2 ± 4.8%, n = 11, *P *< 0.01). Pretreatment with GSPE (6 μg/ml) for 5 min further inhibited AMPA-induced response in the presence of nimodipine (1 μM) (peak 3/peak 1 = 27.3 ± 6.7%, n = 9, *P *< 0.01) (Figure 2B1-B3).

**Figure 3:**
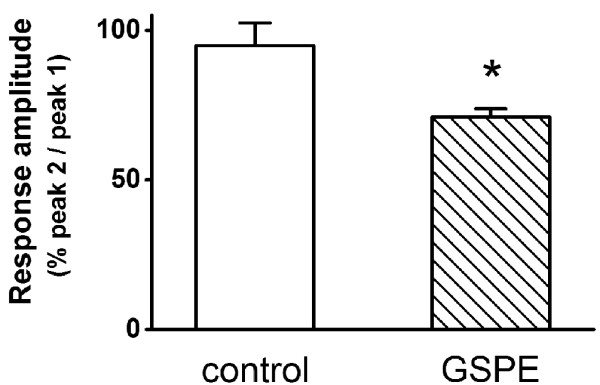
**Effect of GSPE on NMDA-induced [Ca^2+^]_i _response**. Reproducible [Ca^2+^]_i _increase was induced by treatment for 1 min with 100 μM NMDA at 20-min intervals (control, n = 11). Pretreatment with GSPE (6 μg/ml) for 5 min decreased the NMDA-induced response (GSPE, n = 15). Data are expressed as the mean ± SEM. *P < 0.05 relative to NMDA (unpaired Student's *t*-test).

In addition, reproducible NMDA-induced [Ca^2+^]_i _increase was induced by treatment with NMDA (100 μM) for 1 min at 20-min intervals (peak 2/peak 1 = 94.9 ± 7.6%, n = 11). Pretreatment with GSPE (6 μg/ml) for 5 min also significantly inhibited NMDA-induced [Ca^2+^]_i _response (peak 2/peak 1 = 71.1 ± 2.6%, n = 15, *P *< 0.05) (Figure 3).

### Effect of GSPE on high K^+^-induced [Ca^2+^]_i _increase

**Figure 4:**
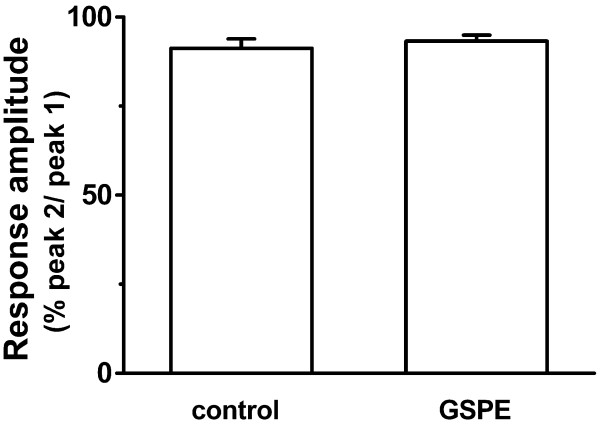
**Effect of GSPE on 50 mM K^+^-induced [Ca^2+^]_i _response**. Reproducible [Ca^2+^]_i _increase was induced by treatment for 1 min with 50 mM K^+ ^HEPES-HBSS at 30-min intervals (control, n = 22). Pretreatment with GSPE (6 μg/ml) for 5 min did not affect the high K^+^-induced [Ca^2+^]_i _response (GSPE, n = 27). Data are expressed as the mean ± SEM.

Binding glutamate to its AMPA receptors induced an influx of Na^+ ^(partly Ca^2+^) into neurons and depolarized the neurons. This depolarization induced secondary activation of voltage-gated Ca^2+ ^channels [23]. To determine the effect of GSPE on glutamate-induced secondary activation of Ca^2+ ^channels, the present study observed whether GSPE affects the depolarization-induced [Ca^2+^]_i _increase by 50 mM K^+ ^HEPES-HBSS (Figure 4). Reproducible [Ca^2+^]_i _increase was induced by treatment for 1 min with 50 mM K^+ ^HEPES-HBSS at 30-min intervals (peak 2/peak 1 = 91.2 ± 2.7%, n = 22). Treatment with GSPE (6 μg/ml) for 5 min did not affect high K^+^-induced [Ca^2+^]_i _response (peak 2/peak 1 = 93.2 ± 1.7%, n = 27, *P *> 0.05) (Figure 4).

### Effect of GSPE on metabotropic glutamate receptor agonist or caffeine-induced [Ca^2+^]_i _increase

**Figure 5:**
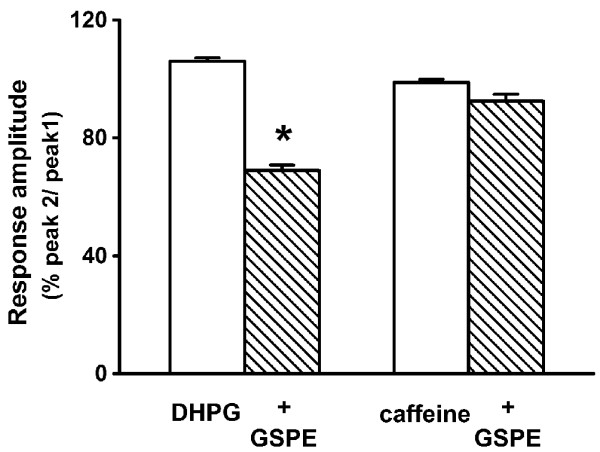
**Effects of GSPE on DHPG- or caffeine-induced [Ca^2+^]_i _response**. Reproducible [Ca^2+^]_i _increase was induced by treatment for 1 min with DHPG (100 μM) at 30-min intervals (DHPG, n = 31). Pretreatment with GSPE (6 μg/ml) for 5 min decreased the DHPG-induced response (+ GSPE, n = 29). Reproducible caffeine-induced [Ca^2+^]_i _increase was induced by treatment for 2 min with DHPG (100 μM) at 10-min intervals (caffeine, n = 14). However, GSPE did not affect caffeine-induced [Ca^2+^]_i _increase (+ GSPE, n = 9). Data are expressed as the mean ± SEM. *P < 0.05 relative to DHPG (unpaired Student's *t*-test).

Group I metabotropic glutamate receptors, composed of mGluR1 and mGluR5, are exclusively expressed at postsynaptic sites in the hippocampus [24]. They are linked to phosphatidylinositol metabolism and the formation of inositol 1,4,5-trisphosphate (IP_3_) and diacylglycerol. Binding of IP_3 _to the IP_3 _receptors initiates release of Ca^2+ ^from intracellular stores [25]. The present study examined whether GSPE affects the metabotropic glutamate receptor agonist response to DHPG-induced [Ca^2+^]_i _increase. Reproducible [Ca^2+^]_i _increase was induced by treatment with DHPG (100 μM) for 1 min at 30-min intervals (peak 2/peak 1 = 105.9 ± 1.3%, n = 31). Pretreatment with GSPE (6 μg/ml) for 5 min significantly inhibited DHPG-induced [Ca^2+^]_i _response (peak 2/peak 1 = 68.9 ± 1.8%, n = 29, *P *< 0.05) (Figure 5).

In addition to IP_3 _receptors, ryanodine receptors can mobilize intracellular Ca^2+ ^stores [26]. Reproducible [Ca^2+^]_i _increase was induced by treatment with caffeine (10 mM) for 2 min at 10-min intervals (peak 2/peak 1 = 98.8 ± 1.0% of control response, n = 14). Pretreatment with GSPE (6 μg/ml) for 5 min did not significantly affect caffeine-induced [Ca^2+^]_i _response (92.5 ± 2.4% of control response, n = 9, P > 0.05) (Figure 5).

### Effect of GSPE on 0.1 mM [Mg^2+^]_o_-induced [Ca^2+^]_i _spikes

**Figure 6:**
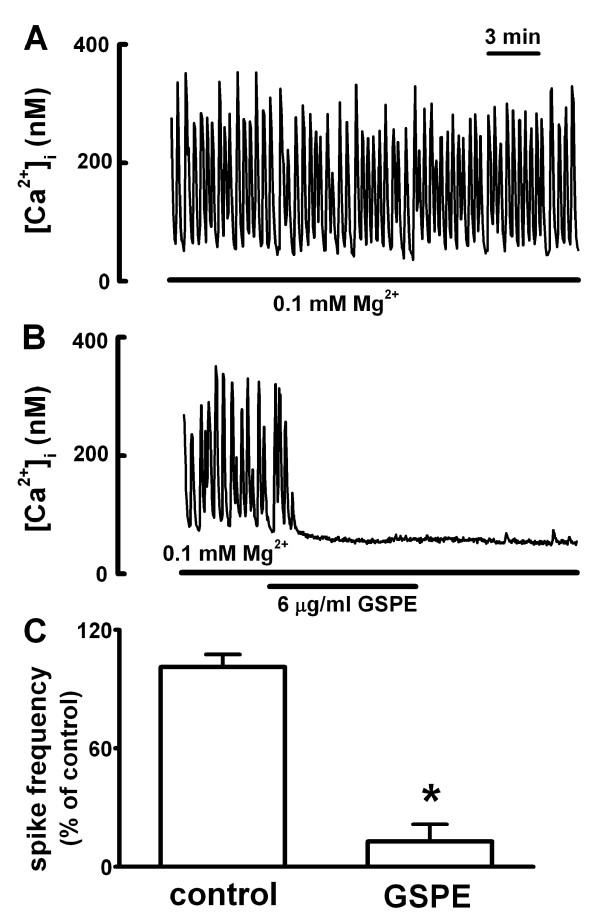
**GSPE inhibits synaptically-mediated [Ca^2+^]_i _spikes induced by treatment with 0.1 mM [Mg^2+^]_o_**. A: Reduction of [Mg^2+^]_o _to 0.1 mM induced [Ca^2+^]_i _spikes. B: Pretreatment with GSPE (6 μg/ml) inhibited 0.1 mM [Mg^2+^]_o_-induced [Ca^2+^]_i _spikes. C: The plot summarizes the inhibition of GSPE on 0.1 mM [Mg^2+^]_o_-induced response (control, n = 8; 6 μg/ml, n = 7). The frequency of [Ca^2+^]_i _spikes was calculated from data collected during a 5 min window before GSPE application for control, and during a 5 min window 5-10 min after application of the drug for GSPE-treated samples. Data are expressed as the mean ± SEM. *P < 0.05 relative to control (unpaired Student's *t*-test).

The next study determined whether GSPE affects synaptically mediated [Ca^2+^]_i _spikes. Previous studies have shown that reducing [Mg^2+^]_o _to 0.1 mM can elicit intense [Ca^2+^]_i _spikes (Figure 6A) which depend on synaptic transmission [27]. [Ca^2+^]_i _spikes were induced by 0.1 mM [Mg^2+^]_o _in the cultured rat hippocampal neurons 13 days after plating. The low [Mg^2+^]_o_-induced [Ca^2+^]_i _spikes gradually disappeared after treatment with GSPE (6 μg/ml). At 10 min after exposure to GSPE, the frequency of [Ca^2+^]_i _spikes was 12.8 ± 8.7% of the initial frequency (Figure 6B &6C).

### Effect of GSPE on 0.1 mM [Mg^2+^]_o_-and glutamate-induced NO formation

**Figure 7:**
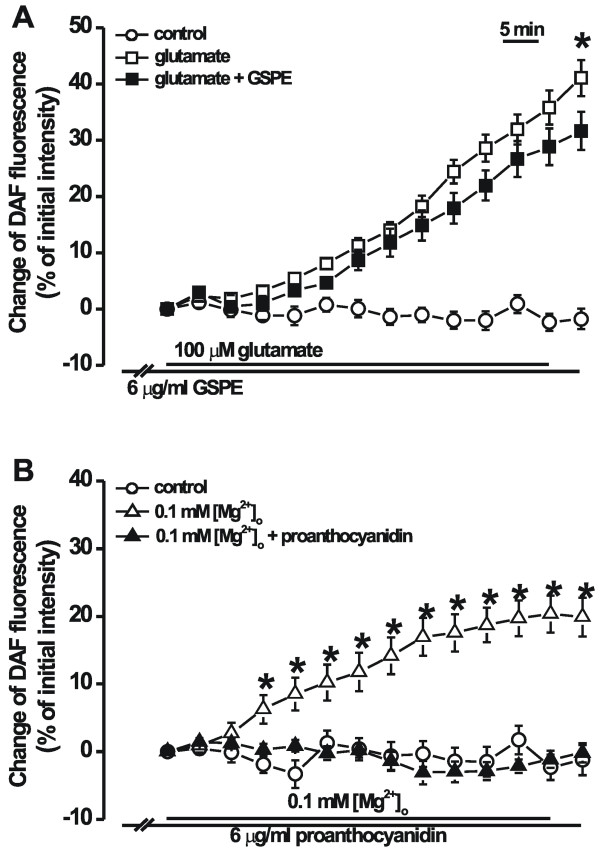
**GSPE decreases glutamate- and 0.1 mM [Mg^2+^]_o_-induced NO formation**. Cells were preincubated with 20 μM DAF-2 DA for 60 min. NO formation was shown as a percentage of the initial intensity of DAF-2T. Treatment with glutamate (100 μM) or 0.1 mM [Mg^2+^]_o _for 1 h significantly increased DAF-2T fluorescence. A: Pretreatment with GSPE (6 μg/ml) decreased the 100 μM glutamate-induced NO formation 65 min after 100 μM glutamate treatment (control, n = 9; 100 μM glutamate, n = 13; 100 μM glutamate + 6 μg/ml GSPE, n = 12). *P < 0.05 relative to 100 μM glutamate (unpaired Student's *t*-test). B: Pretreatment with GSPE (6 μg/ml) decreased the 0.1 mM [Mg^2+^]_o_-induced NO formation 15 min after 0.1 mM [Mg^2+^]_o _treatment (control, n = 10; 0.1 mM [Mg^2+^]_o_, n = 14; 0.1 mM [Mg^2+^]_o _+ 6 μg/ml GSPE, n = 9). Data are expressed as the mean ± SEM. *P < 0.05 relative to 0.1 mM [Mg^2+^]_o _(unpaired Student's *t*-test).

NO is important for glutamate-induced neurotoxicity [12]. The present study determined whether GSPE affects low [Mg^2+^]_o_-induced NO formation using the NO indicator DAF-2DA. Treatment with glutamate (100 μM) or low [Mg^2+^]_o _for 1 h significantly increased DAF-2T fluorescence. While the 0.1 mM [Mg^2+^]_o_-induced increase in NO formation was markedly inhibited by pretreatment with GSPE (6 μg/ml) for 5 min, and the glutamate-induced NO formation was slowly inhibited at a later phase (Figure 7A &7B).

### GSPE protects neuronal cells against 0.1 mM [Mg^2+^]_o_- and oxygen glucose deprivation-induced cell death

**Figure 8:**
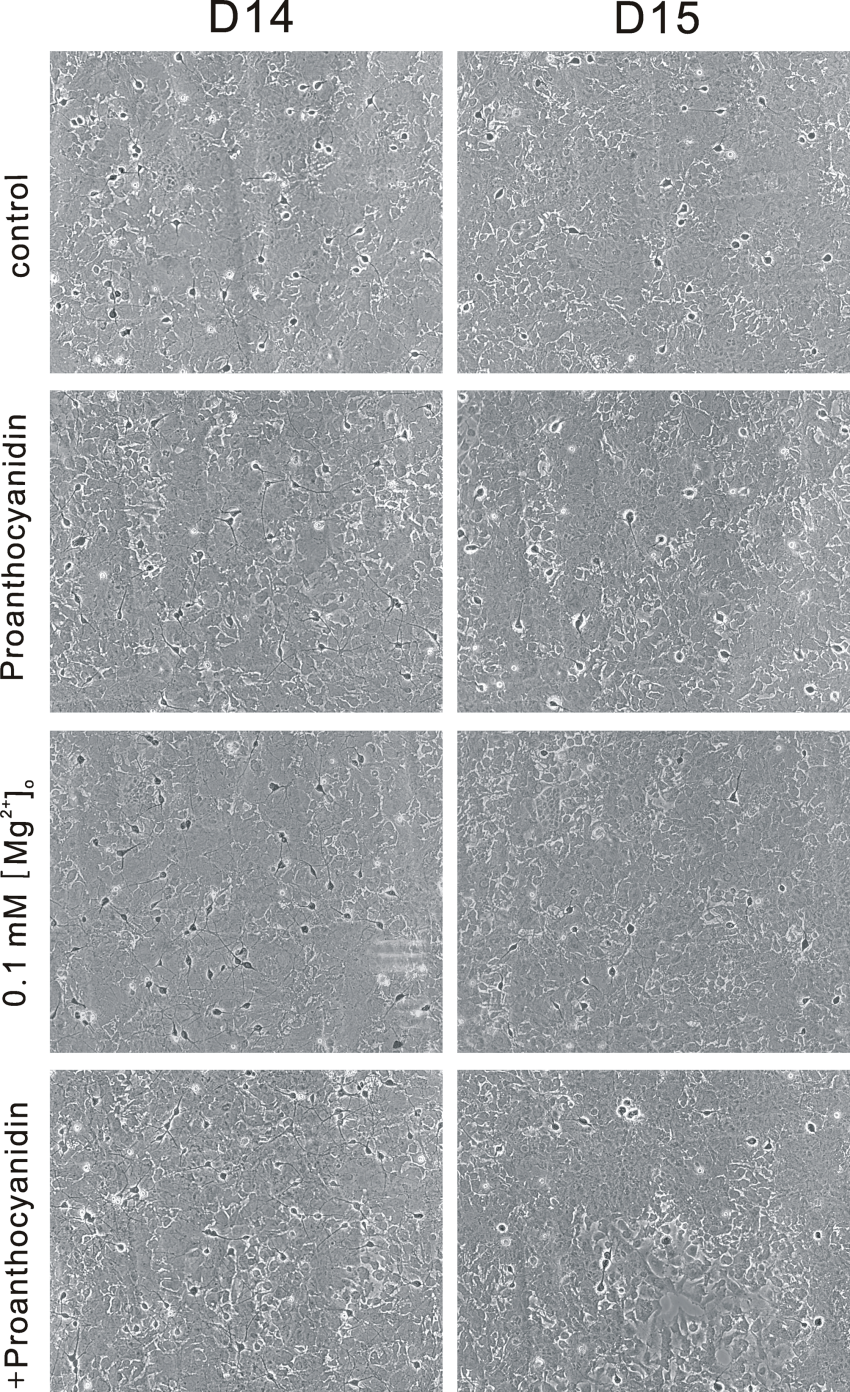
**GSPE protects cells against 0.1 mM [Mg^2+^]_o_-induced neurotoxicity**. Phase-contrast photomicrographs showed the same field of cultured rat hippocampal neurons before treatment (left, D14) and 20-24 h after treatment (right, D15). The cells were treated with normal medium (control), GSPE (6 μg/ml), 0.1 mM [Mg^2+^]_o _and 0.1 mM [Mg^2+^]_o _plus GSPE (+ GSPE)-containing medium at 14 days in culture. Hippocampal neurons (identified by a light halo around the soma and long fine processes) grew on a bed of non-neuronal cells that formed a mosaic beneath them.

**Figure 9:**
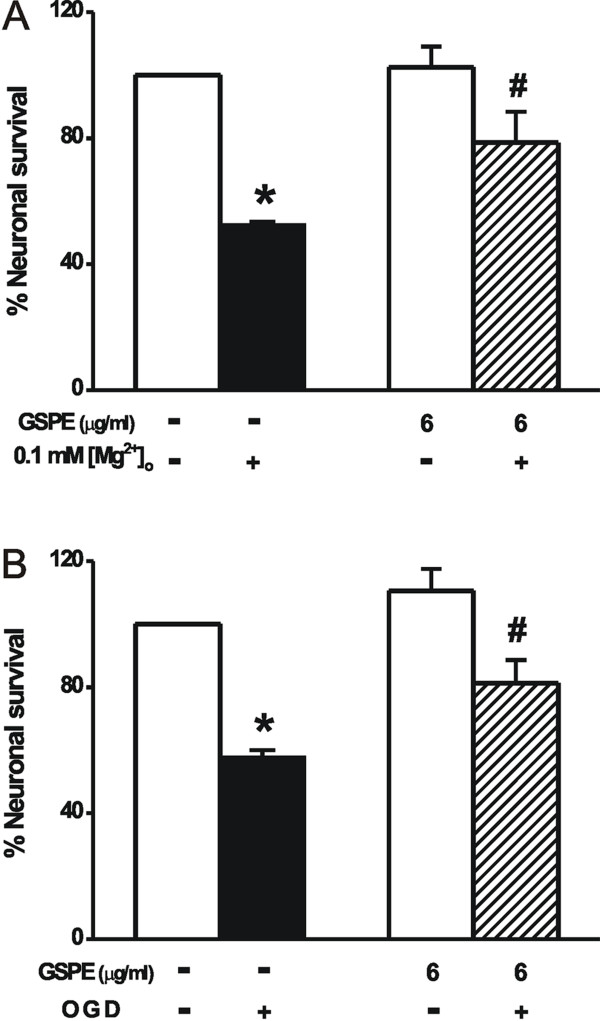
**Effect of GSPE on 0.1 mM [Mg^2+^]_o _and oxygen glucose deprivation-induced neurotoxicity**. A: Hippocampal neurons were exposed to normal medium or 0.1 mM [Mg^2+^]_o_-containing medium with or without GSPE (6 μg/ml, n = 8) for 20-24 h. B: Hippocampal neurons were exposed to normal or oxygen glucose deprived conditions without GSPE (OGD, n = 8) or with GSPE (6 μg/ml, n = 8) for 90 min. After 20-24 h, the same fields of cells were recounted. Data are expressed as the mean ± SEM. *P < 0.001 relative to the control, # *P *< 0.05 relative to 0.1 mM [Mg^2+^]_o _or OGD (ANOVA with Bonferroni's test).

Reduction of [Mg^2+^]_o _in the solution used to bathe cultured CNS neurons elicits an intense pattern of excitatory activity and [Ca^2+^]_i _spikes and causes neuronal cell death [28-30]. The present study was an examination of whether GSPE protects cells against cell death induced by low [Mg^2+^]_o_. Cell viability was determined by counting the number of viable neurons before and 20-24 h after treatment (Figure 8 & Figure 9A). In the control cells, cell survival was decreased slightly (Figure 8, data shown in Methods section). Reduction of [Mg^2+^]_o _markedly decreased neuronal cell survival (52.6 ± 0.9% of the control) (Figure 8 & Figure 9A). A similar proportion of the GSPE-treated cells died relative to the control cells (Figure 8 & Figure 9A). However, a 0.1 mM [Mg^2+^]_o_-induced decrease in cell survival was markedly inhibited by 6 μg/ml GSPE (78.6 ± 9.9% of control) (Figure 8 & Figure 9A). The effect of GSPE on oxygen glucose deprivation-induced cell death was examined further (Figure 9B). The cells in glucose-free BSS, with and without GSPE (6 μg/ml), were gassed with 85% N_2_, 10% H_2_, and 5% CO_2 _for 90 min, and then were regrown in DMEM supplemented with 10% horse serum and penicillin/streptomycin in a CO_2 _incubator for 24 h. Oxygen glucose deprivation decreased neuronal cell survival to 57.8 ± 2.2% of the control. However, treatment with GSPE (6 μg/ml) increased cell survival to 81.3 ± 7.3% of the control.

## Discussion

The present study used an *in vitro *rat hippocampal culture model to determine the inhibitory mechanisms of GSPE in low [Mg^2+^]_o _or oxygen glucose deprivation-induced neuronal cell death. GSPE reduced the glutamate-induced [Ca^2+^]_i _increase by inhibiting the AMPA, NMDA, and DHPG-induced [Ca^2+^]_i _increase in hippocampal neurons. Synaptically mediated low [Mg^2+^]_o_-induced [Ca^2+^]_i _spikes were also inhibited by GSPE. GSPE inhibited low [Mg^2+^]_o _or oxygen glucose deprivation-induced neuronal cell death by inhibition of both [Ca^2+^]_i _increase and Ca^2+^-dependent NO formation.

Glutamate depolarizes membranes by an influx of Na^+ ^(partly Ca^2+^) through non-NMDA receptors, which secondarily activate voltage-gated Ca^2+ ^channels and induce Ca^2+ ^influx [23]. Glutamate also induces Ca^2+ ^influx directly through NMDA receptor channels and Ca^2+^-permeable non-NMDA AMPA receptor channels. In the present study, GSPE inhibited glutamate, AMPA, and NMDA-induced [Ca^2+^]_i _increase, but it did not affect the depolarization-induced [Ca^2+^]_i _increase from 50 mM K^+ ^HEPES-HBSS, suggesting that GSPE inhibits AMPA-induced [Ca^2+^]_i _increase by inhibiting Ca^2+ ^influx directly through Ca^2+^-permeable AMPA receptors. In fact, Ca^2+^-permeable AMPA receptors are strongly expressed in hippocampal neurons, especially early in development [22]. All these data suggested that GSPE inhibited Ca^2+ ^influx through Ca^2+^-permeable AMPA channels and NMDA channels. This data are indirectly supported by other reports that flavonoids such as baicalin, baicalein, and EGCG, decreased glutamate or NMDA-induced [Ca^2+^]_i _increase [15,31].

The group I metabotropic glutamate receptor agonist, DHPG, induces a release of Ca^2+ ^from IP_3_-sensitive stores by activating PLC [25,32]. In the present study, GSPE inhibited DHPG-induced [Ca^2+^]_i _increase. Although the working mechanism of GSPE is not obvious, GSPE may inhibit DHPG-induced Ca^2+ ^release from IP_3_-sensitive stores or DHPG-induced activation of PLC. Therefore, further research is needed to determine whether proanthocyanidin inhibits release of Ca^2+ ^from IP_3_-sensitive stores or metabotropic glutamate receptor-induced activation of PLC.

In the present study, GSPE inhibited glutamate-induced [Ca^2+^]_i _increase by inhibiting AMPA, NMDA, and metabotropic glutamate receptor-induced [Ca^2+^]_i _increase. Reduction of [Mg^2+^]_o _in cultured central nervous system neurons to 0.1 mM elicited [Ca^2+^]_i _spikes that depend on glutaminergic synaptic transmission [27,29,33]. In the present study, GSPE inhibited low [Mg^2+^]_o_-induced [Ca^2+^]_i _spikes. All these data suggest a possibility that proanthocyanidin can inhibit glutaminergic synaptic transmission in hippocampal neurons. In the present study, GSPE did not affect the depolarization-induced [Ca^2+^]_i _increase induced by high K^+^, which is involved in neurotransmitter release in the synaptic terminal. Thus, it is not clear whether proanthocyanidin inhibited synaptic transmission by decreasing glutamate release in presynaptic sites.

In the present study, GSPE completely inhibited low [Mg^2+^]_o_-induced NO formation, and it slightly inhibited glutamate-induced formation. GSPE reportedly has potent inhibitory action on NO production presumably through of the inhibition of Ca^2+^-dependent nitric oxide synthase [34]. In neuronal cells, NO was synthesized from Ca^2+^-dependent enzymes, neuronal nitric oxide synthase [35,36]. Therefore, the inhibition of excessive Ca^2+ ^influx or Ca^2+ ^release from intracellular stores and formation of NO by glutamate in the present study suggest that proanthocyanidin inhibits NO formation by inhibiting glutamate or low [Mg^2+^]_o_-induced [Ca^2+^]_i _increase.

Previous investigations have reported that proanthocyanidin protects multiple target organs from drug- and chemical-induced toxicity. GSPE protects cells against acetaminophen-induced hepato- and nephrotoxicity, amiodarone-induced lung toxicity, doxorubicin-induced cardiotoxicity, and dimethylnitrosamine-induced spleenotoxicity [37]. GSPE inhibited 12-O-tetradecanoylphorbol-13-acetate and O-ethyl-S,S-dipropyl phosphorodithioate-induced brain neurotoxicity [2,37]. Grape seed extract has also been reported to reduce brain ischemic injury in gerbils [4,38] and rats [39], suggesting that the neuroprotective effects of proanthocyanidin are mediated by its antioxidant effects and antiapoptotic effects, respectively. However, there have been no reports on the underlying roles of calcium signalling or NO formation in proanthocyanidin-induced neuroprotection. GSPE inhibited low [Mg^2+^]_o_- and oxygen glucose deprivation-induced neuronal cell death as well as both [Ca^2+^]_i _increase and Ca^2+^-dependent NO formation. Ischemic insults have reportedly induced [Ca^2+^]_i _increase and formation of NO in neurons [10,12,40,41]. In addition, proanthocyanidin blueberry extract is reported to have reversed dopamine, Aβ_42_, and lipopolysaccharide-induced dysregulation of Ca^2+ ^buffering capacity, thereby inducing neuroprotection in hippocampal neurons [20]. These results suggest that proanthocyanidin might inhibit ischemia-induced neuronal cell death by inhibiting glutamate-induced [Ca^2+^]_i _signalling and NO formation as well as antioxidant effects and antiapoptotic effects.

The daily intake of proanthocyanidins may vary from tens to several hundred mg/day depending on diet [42]. Proanthocyanidins, especially oligomeric proanthocyanidins, are more easily absorbed and are present in blood after oral intake [21,43]. Catechin and epicatechin are reportedly bioavailable to the brain after ingestion of oligomeric proanthocyanidin [43], which suggests that oligomeric proanthocyanidins can cross the blood-brain barrier and affect neuronal cells. In fact, the IH636 grape seed proanthocyanidin extract (GSPE) used in the present study was composed of more than 73% oligomeric polyphenolic compounds including monomeric, dimeric, trimeric, and tetrameric proanthocyanidin [44]. Although the biological efficacy of GSPE has been studied previously in humans [37,44], the bioavailablity of GSPE used in the present study remains unknown. However, it should be noted that this particular concentration of grape seed proanthocyanidin extract (GSPE) was less than or equal to the serum concentration in humans following intake of 200 mg/kg proanthocyanidins or oligomeric proanthocyanidins [21]. These data suggest a possibility that IH636 grape seed proanthocyanidin extract (GSPE) can induce neuroprotection after intake of oligomeric proanthocyanidin in humans as well as animals.

## Conclusions

The results of the present study showed that IH636 grape seed proanthocyanidin extract protected neuronal cells against the low [Mg^2+^]_o_- and oxygen glucose deprivation-induced neurotoxicity in cultured rat hippocampal neurons. The neuroprotective effects of proanthocyanidin might have been mediated by inhibition of glutamate-induced calcium signalling and NO formation. These results demonstrated that proanthocyanidin, and especially oligomeric polyphenolic compounds, may have future utility as neuroprotective agents or as supplements against glutamate excitotoxicity-related neurologic disorders such as epilepsy, traumatic brain injury, and ischemia.

## Methods

### Materials

Materials were purchased from the following companies: IH636 grape seed proanthocyanidin extract (GSPE) from InterHealth Nutraceuticals (Benicia, CA, USA); Dulbecco's modified Eagle's medium (DMEM) and fetal bovine serum (FBS) from Invitrogen (Carlsbad, CA, USA); fura-2 acetoxymethyl ester (AM) from Molecular Probes (Eugene, OR, USA); 4,5-diaminofluorescein diacetate (DAF-2DA) from A.G. Scientific (San Diego, CA, USA); N-methyl-D-aspartate (NMDA), alpha-amino-3-hydroxy-5-methyl-4-isoxazolepropionic acid (AMPA), (S)-3,5-dihydroxyphenylglycine (DHPG) and all other reagents from Sigma (St. Louis, MO, USA).

### Primary rat hippocampal cell culture

Rat hippocampal neurons were grown in primary culture as previously described [45] with minor modifications. Adult maternal Sprague-Dawley rats (250-300 g) were used in the present study. All experimental procedures performed on the animals were conducted with the approval of the Catholic Ethics Committee of the Catholic University of Korea and were in accordance with the National Institutes of Health *Guide for the Care and Use of Laboratory Animals *(revised 1996). Fetuses were removed on embryonic day 17 from maternal rats anesthetized with urethane (1.3 g/kg b.w., i.p.). Hippocampi were dissected and placed in Ca^2+- ^and Mg^2+^-free Hank's balanced salt solution, pH 7.4. Cells were dissociated by trituration through a 5-ml pipette and then a flame-narrowed Pasteur pipette. Cells were pelleted and resuspended in Dulbecco's modified Eagle's medium (DMEM) without glutamine and supplemented with 10% fetal bovine serum and penicillin/streptomycin (100 U/ml and 100 μg/ml, respectively). Dissociated cells were then plated at a density of 50,000 cells/well onto 25-mm-round cover glasses that were coated with poly-L-lysine (0.1 mg/ml) and washed with H_2_O. The cells were grown in a humidified atmosphere of 10% CO_2_-90% air (pH 7.4) at 37°C. The medium was replaced 72-90 h after plating with DMEM supplemented with 10% horse serum and penicillin/streptomycin and fed every 7 days by exchange of 25% of the medium. The cells were cultured without mitotic inhibitors for a minimum of 12 days. The cells were used after 14-15 days in culture. During this period, neurons developed extensive neuritic networks, and formed functional synapses.

### Digital [Ca^2+^]_i _imaging

To measure [Ca^2+^]_i_, hippocampal cells were incubated in 4 μM fura-2 AM in HEPES-buffered Hank's salt solution (HHSS: 20 mM HEPES, 137 mM NaCl, 1.3 mM CaCl_2_, 0.4 mM MgSO_4_, 0.5 mM MgCl_2_, 0.4 mM KH_2_PO_4_, 0.6 mM Na_2_H_2_PO_4_, 3.0 mM NaHCO_3_, and 5.6 mM glucose) containing 0.5% bovine serum albumin for 45 min at 37°C. The cover glass was then mounted in a flow-through chamber that was superfused at a rate of 1.5 ml/min. Digital calcium imaging was performed as described by Rhie et al. [46]. The chamber containing the fura-2-loaded cells was mounted on the stage of an inverted microscope (Nikon TE300, Tokyo, Japan), and alternately excited at 340 nm and 380 nm by rapidly switching optical filters (10 nm band pass) mounted on a computer-controlled wheel (Lambda 10-2, Sutter Instruments Inc., Novato, CA, USA) placed between a 100 W Xe arc lamp and the epifluorescence port of the microscope. Excitation light was reflected from a dichroic mirror (400 nm for fura-2) through a 20× objective (Nikon; N.A. 0.5). Digital fluorescence images (510 nm, 40 nm band-pass) were collected with a computer-controlled, cooled, charge-coupled device camera (1280 × 1035 binned to 256 × 207 pixels, Quantix, Photometrics, Tucson, AZ., USA). Image pairs were collected every 2-20 s using an Axon Imaging Work Bench 2.2 (Axon Instruments, Inc., Forster City, CA., USA); exposure to excitation light was 120 ms per image. [Ca^2+^]_i _was calculated from the ratio of the background-subtracted digital images. Cells were delimited by producing a mask that contained pixel values above a certain threshold applied to the 380 nm image. Background images were collected at the beginning of each experiment after removing cells from another area to the coverslip. Autofluorescence from cells not loaded with the dye was less than 5% and thus not corrected. Ratio values were converted to free [Ca^2+^]_i _by the equation [Ca^2+^]_i _= K_d_β(R-R_min_)/(R_max_-R), in which *R *was the 340/380 nm fluorescence emission ratio and *K_d _*= 224 nM was the dissociation constant for fura-2. R_min_, R_max_, and β was determined in ionomycin-permeabilized cells in calcium-free and saturated solutions (R_min_= 0.325, R_max_= 9.23, β = 7.61).

### [Ca^2+^]_i _measurement using fura-2-based-photometry

[Ca^2+^]_i _spikes were measured using fura-2-based-microfluorimetry [45]. The chamber containing the fura-2-loaded cells was mounted on an inverted microscope (Nikon S-100F, Nikon, Tokyo, Japan). For the excitation of fura-2, light from a 75 W Xe arc lamp (LPS-220, Photon Technology International, NJ, USA) was passed through band-pass filters (340/20 and 380/20 nm, respectively). Excitation light was reflected sequentially from a dichroic mirror (400 nm) through a 40× phase contrast oil immersion objective (Nikon, Tokyo, Japan). Emitted light was reflected through a 510 nm filter to a photomultiplier tube (Model 710, Photon Technology International, NJ, USA) operating in photon-counting mode. Recordings were defined spatially with a rectangular diaphragm (D-104C, Photon Technology International, NJ, USA). [Ca^2+^]_i _spikes were induced by HHSS containing 0.1 mM MgCl_2 _and 10 μM glycine. [Ca^2+^]_i _was calibrated by the same method that was used for the digital [Ca^2+^]_i _imaging. R_min_, R_max_, and β were 0.86, 14.89, and 7.42, respectively.

### Measurement of nitric oxide (NO)

To measure the formation of NO, the cells were incubated in an NO indicator DAF-2DA (20 μM) in HHSS without BSA for 60 min at 37°C. After DAF-2DA loading, the cells were rinsed with HHSS for 10 min and placed in a flow-through chamber. DAF-2T (the fluorescent triazolofluorescein produced by NO and DAF-2 reaction) images were obtained through excitation at 480 nm and emission at 535 nm/25 nm (DM 505 nm) [47] after treatment with or without GSPE.

### Toxicity

For toxicity experiments, cells were plated on microetched coverslips (Belco Biotechnology, Vineland, NJ, USA) and at least 100 neurons were counted. In 0.1 mM Mg^2+ ^medium-induced excitotoxicity experiments, coverslips were exposed for 24 h to the 0.1 mM Mg^2+ ^medium with or without GSPE at 14 days in culture. After 20-24 h, the same fields of cells were recounted. In oxygen glucose deprivation-induced excitotoxicity experiments) [48], cultures were washed 3 times with a balanced salt solution (BSS: 116 mM NaCl, 5.4 mM KCl, 0.8 mM MgSO_4_, 1.0 mM NaH_2_PO_4_, 26.2 mM NaHCO_3_, 1.8 mM CaCl_2_, and 10 mg/L phenol red) lacking glucose and were aerated with an anaerobic gas mix (95% N_2_/5% CO_2_) for 10 min to remove residual oxygen, then were transferred to an anaerobic chamber (1025/1029 Anaerobic System, ThermoForma, Ohio, USA) containing a gas mixture of 5% CO_2_, 10% H_2_, and 85% N_2 _for 90 min. To terminate the oxygen glucose deprivation, cells were removed from the anaerobic chamber and then carefully washed with DMEM supplemented with 10% horse serum and penicillin/streptomycin. After 20-24 h, the same fields of cells were recounted.

Viable neurons were identified based on morphological criteria; they were phase-bright, had rounded somata, and extended long fine processes. Cell death was determined by comparing the number of viable neurons before and after treatment [30,49]. Viable neurons obtained were normalized and expressed as a percentage of sham-treated sister cultures (defined as 100%). Control experiments showed that the loss of viable neurons assessed in this manner was proportional to the number of neurons damaged. In control cells (medium exchange only), 28.4 ± 1.5% of the cells in the 0.1 mM Mg^2+ ^experiment (Figure 8) and 27.3 ± 1.2% of the cells in the OGD experiment died.

### Statistical analysis

Data are expressed as the mean ± SEM. Significance was determined using a Student's *t*-test or one-way analysis of variance (ANOVA) followed by a Bonferroni test.

## Lists of Abbreviations

AM: acetoxymethyl ester; AMPA: alpha-amino-3-hydroxy-5-methyl-4-isoxazolepropionic acid; [Ca^2+^]_i_: intracellular free Ca^2+ ^concentration; DAF-2DA: 4,5-diaminofluorescein diacetate; DHPG: (*RS*)-3,5-Dihydroxyphenylglycine; DMEM: Dulbecco's modified Eagle's medium; EGCG: (-)-epigallocatechine gallate; FBS: fetal bovine serum; GSPE: grape seed proanthocyanidin extract; HHSS: HEPES-buffered Hank's salt solution; IP3: inositol 1,4,5-trisphosphate; [Mg^2+^]_o_, extracellular Mg^2+ ^concentration; NMDA: N-methyl-D-aspartate; NO: nitric oxide; NOS: nitric oxide synthase; OGD: oxygen glucose deprivation; PLC: phospholipase C

## Authors' contributions

SHY designed the study. S-HA, HJK, IJ, YJ H, M-JK, D-JR and SJH performed the experiments and analyses. S-HA, HJK, Y-HJ, and SHY wrote the paper. All authors read and approved the final manuscript.
